# Impact of Patient Navigation on Population-Based Breast Screening: a Systematic Review and Meta-analysis of Randomized Clinical Trials

**DOI:** 10.1007/s11606-022-07641-y

**Published:** 2022-06-01

**Authors:** Lu Tian, Lei Huang, Jie Liu, Xia Li, Aisha Ajmal, Maryam Ajmal, Yunjin Yao, Li Tian

**Affiliations:** 1 The Third Central Clinical College of Tianjin Medical University, Tianjin, 300170 China; 2 Tianjin Key Laboratory of Extracorporeal Life Support for Critical Diseases, Tianjin, China; 3 Artificial Cell Engineering Technology Research Center, Tianjin, China; 4 Tianjin Institute of Hepatobiliary Disease, Tianjin, China; 5 The 3rd Department of Breast Cancer, China Tianjin Breast Cancer Prevention, Treatment and Research Center, Tianjin Medical University Cancer Institute and Hospital, National Clinical Research Center of Cancer, Tianjin, China; 6 Department of Heart Center, Tianjin Third Central Hospital, Tianjin, 300170 People’s Republic of China; 7 Department of Clinical Laboratory, Tianjin Third Central Hospital, Tianjin, 300170 China; 8 St George’s Hospital Medical School, St George’s, University of London, London, SW17 0RE UK; 9 GKT School of Medical Education, Faculty of Life Science and Medicine, King’s College London, London, SE1 1UL UK; 10 Department of Thyroid Disease, The First Affiliated Hospital, Zhejiang University School of Medicine, Hangzhou, China; 11 The Third Central Hospital of Tianjin, 83 Jintang Road, Hedong District, Tianjin, 300170 China

**Keywords:** patient navigation, early detection of cancer, barriers to care, health services research

## Abstract

**Background:**

Unsatisfactory cancer screening results are often associated with poor prognosis. This study synthesized the literatures addressing the impact of patient navigation (PN) interventions on population-based breast cancer screening promotion to identify characteristics of the model for addressing breast cancer disparities.

**Methods:**

We searched Pubmed, Embase, Web of Science, and the Cochrane Central Registry from inception to 31 December 2020 for randomized controlled trials (PROSPERO: CRD42021246890). We double blindly abstracted data and assessed study quality. We assessed screening completion rates and diagnostic resolution using random-effects models between those receiving navigation and controls.

**Results:**

Of 236 abstracts identified, 15 studies met inclusion criteria. Nine of the papers evaluated the impact of PN on breast screening, while the other six were on the resolution of abnormal screening results. Compared to the non-PN group, PN improved screening completion (OR: 2.0, 95% CI: 1.4–2.8]) and shortened the time to diagnosis (WMD: − 9.90 days, 95% CI: − 19.09 to − 0.71).

**Conclusions:**

Patient navigation improves breast cancer screening rates but does not improve resolution of abnormal tests.

**Supplementary Information:**

The online version contains supplementary material available at 10.1007/s11606-022-07641-y.

## INTRODUCTION

Mortality due to breast cancer remains high globally. Two causes include low participation in screening and delays in diagnosis.^1^ Early detection and treatment reduce breast cancer death.^2^ However, in some specific populations, socio-economic factors may be an obstacle to participation in population-based breast screening.

Patient navigation (PN) has emerged as one partial solution to reduce disparities in cancer care delivery. Trained navigators can promote cancer screening, follow-up of abnormal tests, and timely treatment.^3, 4^ However, the literature on navigator effectiveness is mixed^5–7^ and interpreting these studies is difficult, owing to considerable heterogeneity. Two recent systematic reviews including both RCTs and observational studies concluded that PN improves the screening rate of many tumors, including breast cancer.^8, 9^ However, these studies did not analyze the impact of PN on the diagnosis rate of abnormal screening findings. Reduction in breast cancer mortality requires that abnormal mammograms be followed by a process that leads to timely definitive diagnosis and treatment.^10^ Unfortunately, this follow-up process was shown to be incomplete or delayed in some vulnerable populations. Our systematic review aims to assess the impact of patient navigation on screening and resolution of abnormal findings for breast cancer.

## PATIENTS AND METHODS

We followed PRISMA guidelines in conducting our review^11^ and registered our protocol with PROSPERO (CRD42021246890).

### Search Strategy and Study Selection

The three authors independently reviewed results from our search of PUBMED, EMBASE, Web of Science, and Cochrane Central Register of Controlled Trials databases from their date of inception to 31 December 2020. The search strategy recommended by a librarian is provided in the Supplementary data. Both unpublished and published studies were eligible for inclusion. After removing the duplicate literature, three authors (Lu T., L. H., and Y. Y.) screened the titles and abstracts of the search results. The final inclusion decision was based on the independent review of the full text by the three authors. Any discrepancies arising from the process should be settled by consensus.

### Eligibility Criteria

Our review was limited to randomized trials of PN among female participants over 18 years old and not pregnant. Studies were excluded if any of the following conditions occurred: (1) participant(s) had a history of cancer or were receiving anti-tumor treatment; (2) participant(s) had received PN intervention in the past; (3) participant(s) were/are living in a pension institution; (4) participant(s) had a history of mastectomy; (5) the screening mode was not community-based; (6) interventions did not meet the definition of PN, such as only phone-call or email reminder; (7) data were insufficient to obtain OR and 95% CI for outcome; (8) full texts were unavailable; and (9) the types of literature comprised abstract, letter, review, protocol, conference presentations, editorials, and/or expert opinions. When the same or partially identical cohorts were reported in different published studies, the most comprehensive study was selected.

### Data Extraction

For all studies eventually included in the meta-analysis, study characteristics were independently extracted using a standard data extraction form by each of the three authors (Table 1). Again, discrepancies were resolved by consensus. For studies that provided graphs of time to DR by days from initial screening instead of mean and SD, we used the Engauge-Digitizer software (version 11.2) to obtain the approximate number of people diagnosed at a specific time point, so as to reasonably estimate the diagnostic time. For studies that provided data by BI-RADS group or race, we pooled the data to get overall effect measures.

**Table 1:** Summary Characteristics of Included Studies

Study	Race/ethnicity	Sample size	Follow-up (months)	Age^*^	Primary language	Income	Education	Screening or diagnosis method	Outcome	Navigation type
Nuño,^13^ 2011, USA	Hispanic: 100%	371	24	60.3 ± 8.38	Spanish 90.5%, English 6.5%, both 3%	$914.05 ± 473.04/month	High school and less 95%, some college or more 5%	Mammogram	Self-reported receipt of mammography screening	A trained promotor presented a 2-h group session as an educational intervention and provided referral information about community resources for cancer screening and health care in general
Ginsburg, ^14^ 2014, Bangladesh	Bangladesh: 100%	14,510	4	38.35 ± 11.5	Not available	Household monthly expenditure: ≤ $115 79.1% , > $115 11.3%, missing 9.6%	High school and less 90.2%, missing 9.8%	Abnormal CBE	No. with abnormal CBE	Smartphone with applications to guide interview, report data, show motivational video, and offer an appointment for women with an abnormal CBE and a community health worker
Marshall,^16^ 2016, USA	Black: 100%	1358	45.6	≥ 65	Not available	Annual household income < $20,000 53.5%, ≥ $20,000 46.5%	High school and less 54.0%, > high school diploma 46.0%	Mammogram	Self-reported receipt of mammography screening	Printed educational materials + patient navigation services
Atlas,^16^ 2014, USA	White: 76%, others: 24%	46,953	12	42–74	English 89.6%	Not available	Not available	Mammogram	Average cancer screening rates	Web-based IT application and intense outreach including frequently attempting contact, exploring individual barriers to screening, educating patients, providing reminder calls, arranging transportation, assisting with visit preparation, and accompanying patients to visits
Percac-Lima,^17^ 2016, USA	White: 64%, others: 36%	1612	8	57 ± 9.35	English 72.8%	Low income	Not available	Mammogram	Average cancer screening rates	Navigators used the IT system to track these patients, contact them in their own language, and provide intense outreach to help them complete cancer screening.
Phillips,^18^ 2011, USA	White: 29%, other: 71%	3895	24	60 ± 5 (51–70)	English 77%, Spanish 7%, others 16%	Not available	High school and less 63% , some college/voc/tech program 18%, graduated college/post-grad 15%	Mammogram	Mammography completion rate	Navigators completed a series of a telephone calls or sent letters to inform women of their need for a mammogram and the availability of the navigator to support them, inquired about individual barriers to accessing care, and then utilized available resources to address those barriers.
Maxwell,^19^ 2010, USA	Asian (Korean): 100%	176	6	52 ± 8 (40–73)	Korean	Annual household income < $20,000 58%, > $20,000 42%	High school and less 52%, college 47%	Mammogram, ultrasound biopsy, CBE, etc.	Self-reported completion of the recommended follow-up exam	An English–Korean bilingual navigator provided individually tailored navigation including reminding women before an appointment, explaining the need for and the nature of the diagnostic follow-up exam, meeting women at the referral clinic, helping them to complete forms, and providing information and emotional support
Braun,^20^ 2015, USA	Hawaiian: 45%, Filipino: 35%, Japanese: 11%, other: 8%	260	12	67.5	English 82.2%	Not available	High school and less 68.2, > high school 31.8%	Mammogram	Mammography completion rate	Navigators performed tasks including outreach, education, making appointments, sending reminders, providing transportation to appointments, communicating with providers, and completing paperwork.
Slater,^21^ 2018, USA	White 68%, others 32%	22,113	3	58.8 ± 6.35	English 83%, others 17%	Above low income 41.1%, low income 42.8%, missing 16.1%	High school or less 85.6, more than high school 14.4	Mammogram	Mammography completion rate	Mailers prompted recipients to call a toll-free number and provided callers with support and guidance related to barriers to cancer screening and care.
Bastani,^5^ 2010, USA	Hispanic: 76%, non-Hispanic: 24%	1671	6	< 40 27%, 40–49 32%, > 50 42%	Not available	Low income	Not available	Medical record	Diagnostic resolution rates	Provided women with information on breast abnormalities and help with emotional and concrete barriers to attendance at follow-up appointments. The calls focused on how to navigate the complex county health system, fee schedules, waiver of payment, and appointment scheduling.
Ferrante,^22^ 2008, USA	Black: 59.0%, Hispanic: 27.6%, other: 13.3%	105	2	50.1 ± 11.6	Not available	Low income	High school and less 76.2%, > high school 23.8%	Biopsy (pathology)	Diagnostic resolution rates/time to diagnostic resolution	Provided patients with emotional and social support; helped patients make appointments and arrive at scheduled appointments on time and prepare, facilitated applications for financial assistance; connected patients with resources and support systems; and facilitated interaction and communication with healthcare staff and providers.
Battaglia,^6^ 2012, USA	White: 37%, other: 63%	715	12	18–40 12%, 41 + 64 75%, > 65 13%	English 61%, Spanish 20%, other 19%	Low income	Not available	BI-RADS 0 ultrasound, BI-RADS 4–5 biopsy, BI-RADS 3, mammogram	Time to diagnostic resolution	Identified barriers to recommended care, and developed strategies to address these barriers, with the focus on timely completion of the diagnostic evaluation. Follow-up occurred by telephone, by mail, and in face-to-face meetings, usually at the health center.
Raich,^24^ 2012, USA	White: 24%, other: 76%	628	12	Not available	English 67%, Spanish 30%	< $20,000 76%	High school and less 66%, > high school 32%	Biopsy (pathology)	Diagnostic resolution rates/time to diagnostic resolution	Identified and assessed practical barriers, social support, and intention to complete the recommended course of care. They ensured that the required examinations were scheduled and communicated with clinic staff regarding patient needs and concerns. They accompanied patients to their appointments when fear, social support, and language barriers were identified, or if requested.
Hoffman,^28^ 2012, USA	White: 8.0%, other: 91.9%	864	12	18–98	Not available	Not available	Not available	Biopsy (pathology)	Time to diagnostic resolution	Not available
Dudley,^23^ 2012, USA	White: 33.2%, other: 53.6%	461	2	49.6 vs 52	English 65.5%, Spanish 32.3%, other 2.2%	< $20,000 68.1%	High school and less 66.7%, > high school 31.5%	Mammogram/pathology (if cancer)	Diagnostic resolution rates/time to diagnostic resolution	Intake, assessment, analysis of needs, development of a care plan, implementing the plan, tracking, and evaluation

*CBE* clinical breast examination

*Years, range or mean ± SD

### Risk-of-Bias Appraisal

We assessed literature quality using the Cochrane Risk of Bias tool.^25^ Review Manager (v 5.3.5) was used to generate “risk of bias” graph and summary.

### Statistical Analysis

We performed statistical analyses using Stata/SE (College Station, TX, v13.0) and the meta package in R (version 3.4.3). We calculated ORs and their associated 95% CIs to assess outcomes and considered a *p* value less than 0.05 to be statistically significant. A significant degree of heterogeneity between studies was defined as both the *I*^2^ statistic with a cut off of ≥ 50%, and the *χ*^2^ test with a *p* value < 0.10.^26^ Effect size was calculated using random-effects models.^27^ We assessed heterogeneity using subgroup analyses (Stata), sensitivity analysis (Stata), and meta regression (R). The criteria of grouping in subgroup analysis was established based on clinical significance and overall data distribution. Publication bias was assessed by Egger’s test with visual inspection of funnel plots (Stata).^12^ We assessed the potential impact of publication bias using the Trim and Fill approach (Stata).^12^

## RESULTS

### Study Characteristics

**Figure 1:**
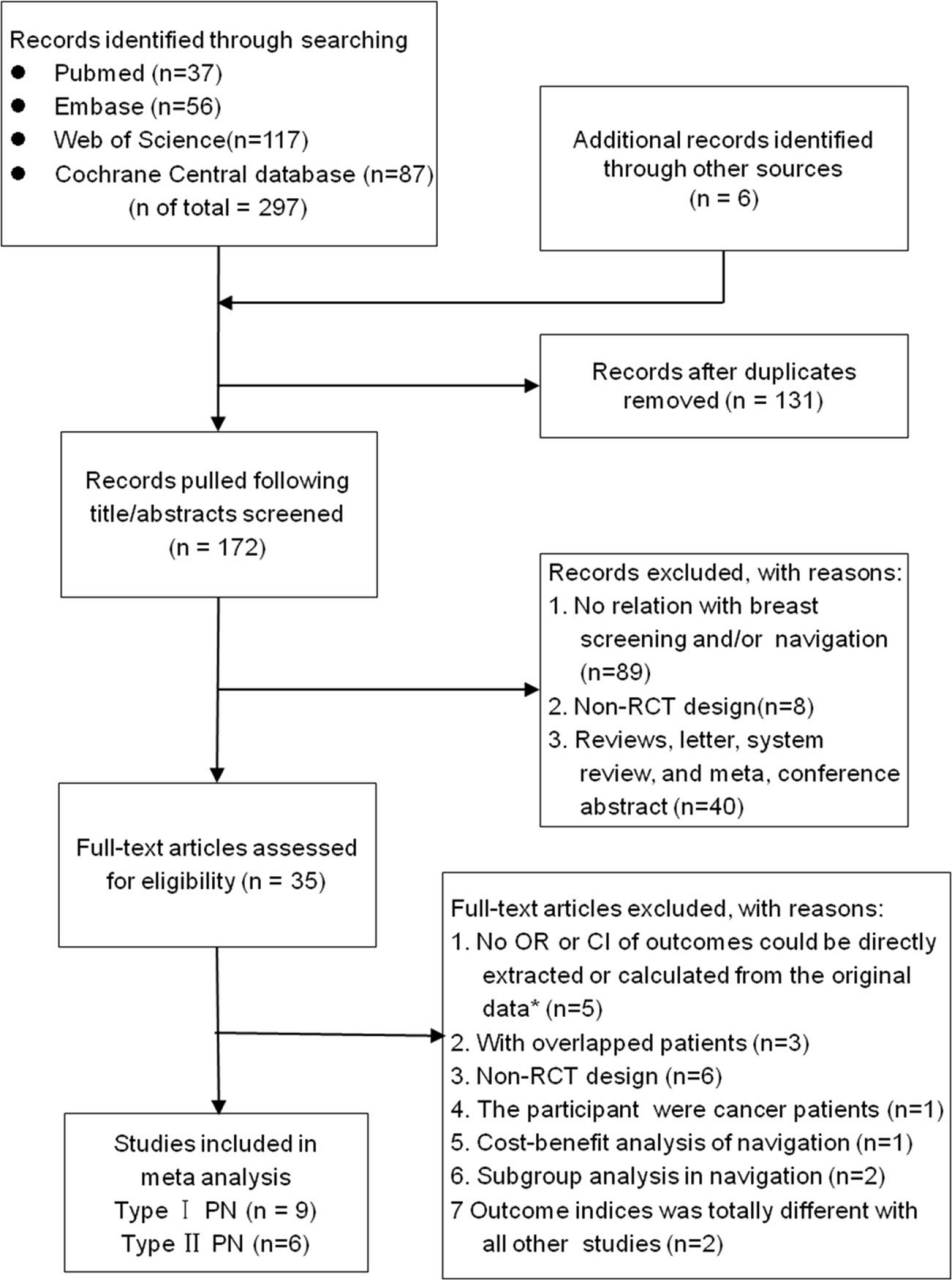
Search strategy and final included and excluded studies.

**Figure 2:**
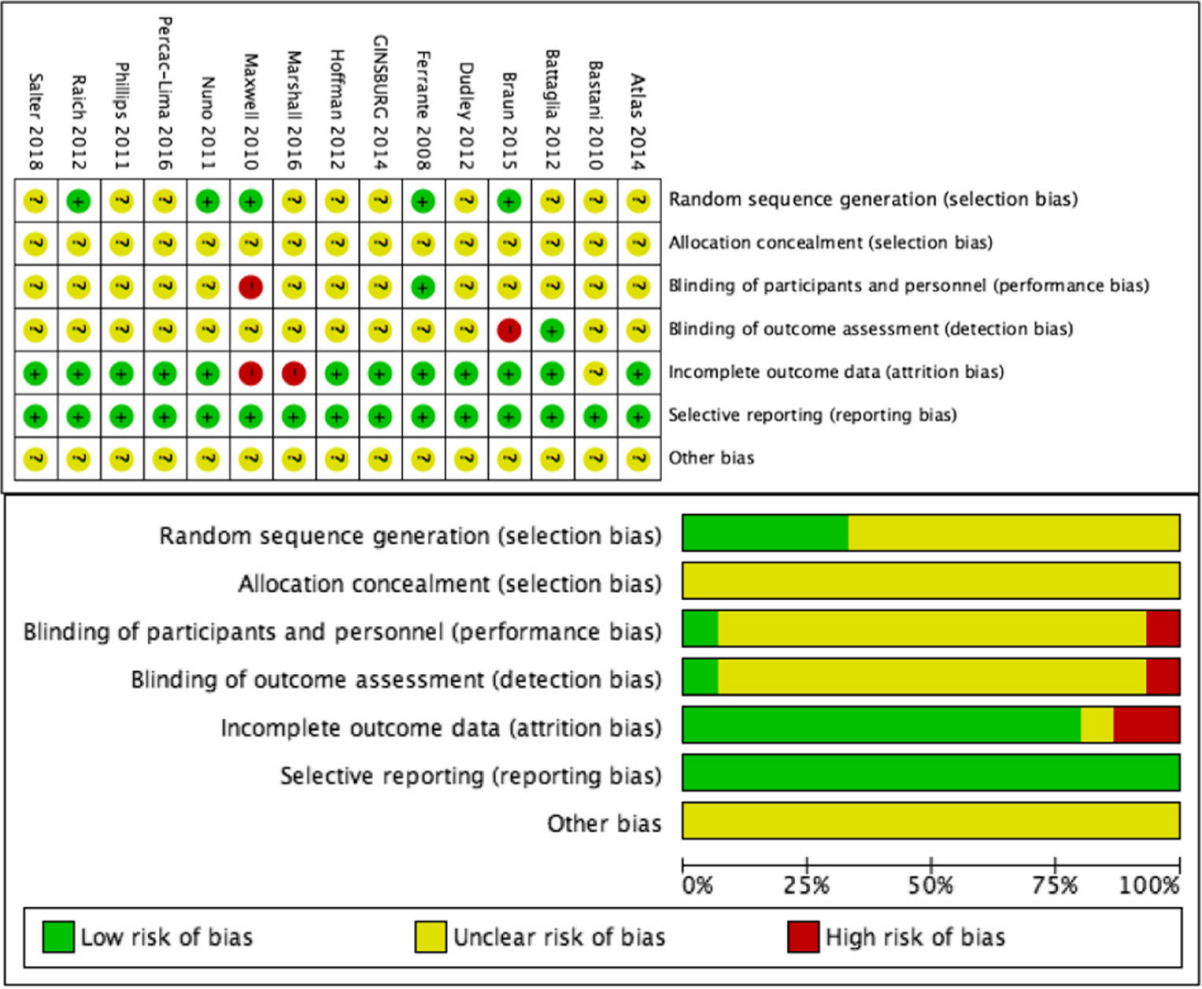
Quality evaluation of the included literature based on the Cochrane Collaboration Network bias risk assessment tool.

We included 9 papers^13–21^ on the impact of PN on screening and six papers^5, 6, 22–24, 28^ on diagnostic resolution (Fig. 1). Study characteristics are presented in Table 1. A considerable number of trials had unclear risk of bias on some measures, suggesting only modest study quality (Fig. 2). Three trials on screening^15,^ ^19,^ ^20^ were judged to be at high risk of bias. All trials enrolled were partially or fully sponsored by the government.

### Effect of PN on Completion of Breast Screening

**Figure 3:**
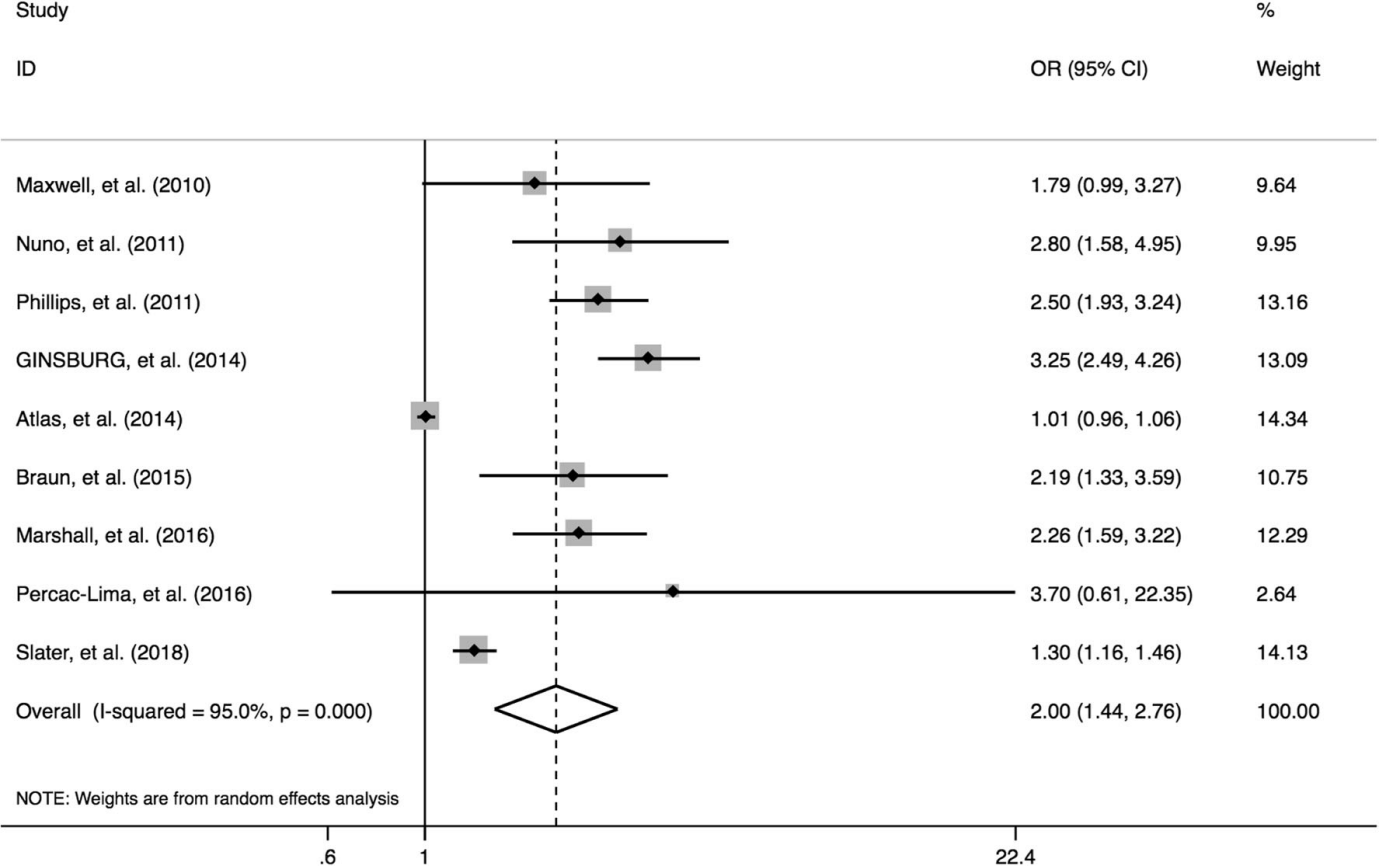
Forest plot of screening completion rate of trials evaluating navigation care.

PN improved the likelihood of completing breast cancer screening (OR: 2.0, 95% CI: 1.4–2.8, *I*^2^ = 95.0%, Fig. 3). Sample size (*p* < 0.01), race (*p* < 0.01), and education level (*p* < 0.01) contributed to heterogeneity, explaining 65%, 86%, and 71% respectively.

In our sensitivity analysis, we found that no single study was overly influential. Our results were not significantly changed by excluding trials at high risk of bias (OR: 1.95, 95% CI: 1.3–2.9, *I*^2^ = 96.3%).

We found evidence of publication bias (Egger’s test, *p* = 0.006), though the effect was still significant after adjusting for publication bias (OR 1.7, 95% CI: 1.3–2.2).

### Effect of PN on Diagnostic Resolution

**Figure 4:**
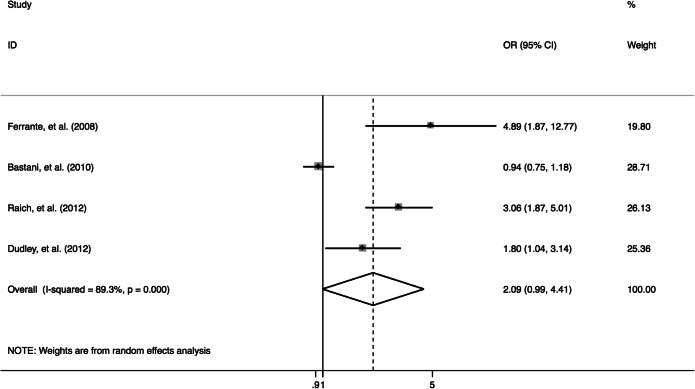
Forest plot of diagnosis resolution rate of trials evaluating navigation care.

**Figure 5:**
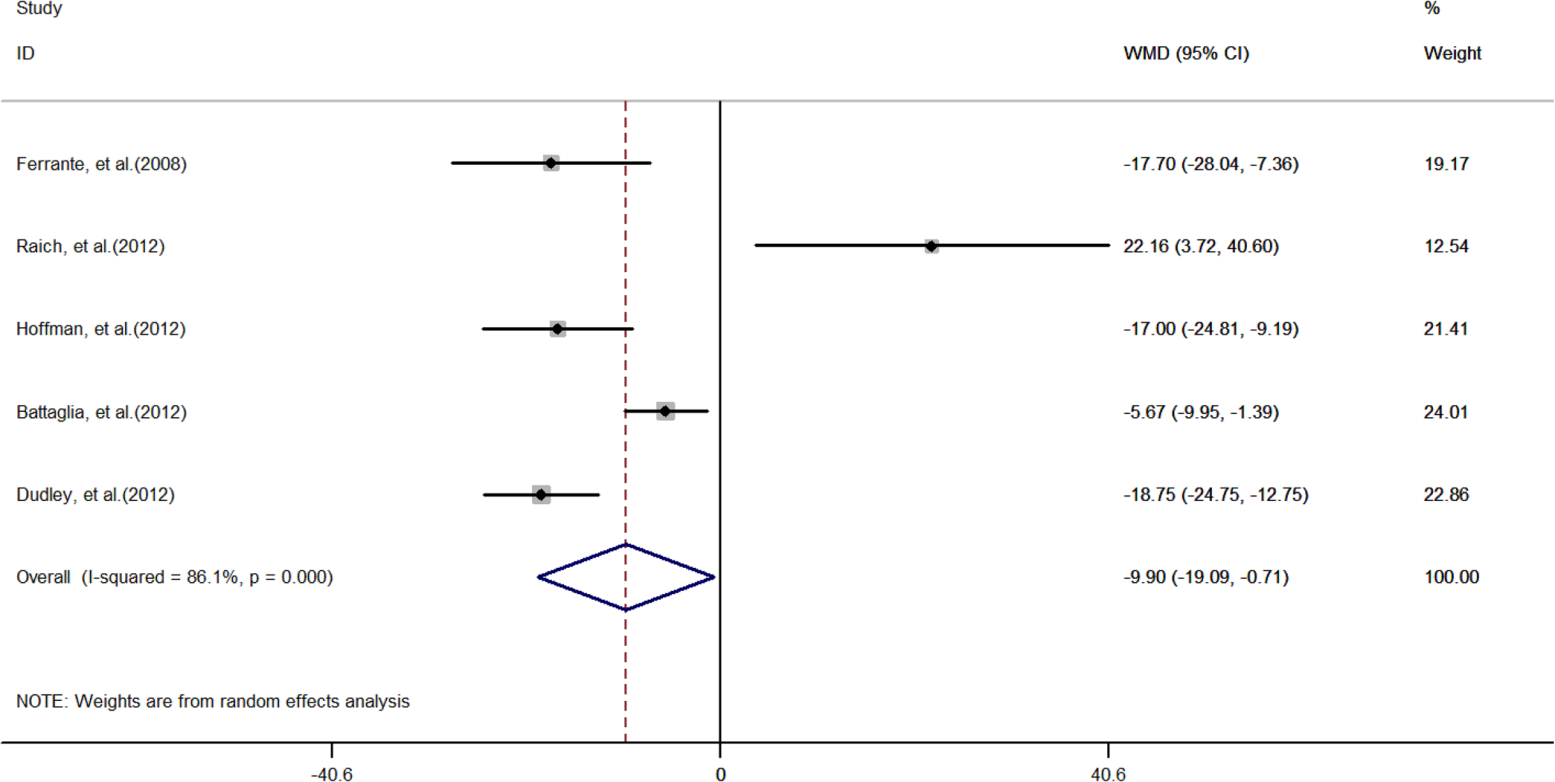
Forest plot of diagnosis resolution time of trials evaluating navigation care.

Patient navigation did not significantly improve diagnostic resolution (OR: 2.1, 95% CI: 0.99–4.4, *I*^2^ = 89.3%, Fig. 4), though it reduced the average time until diagnostic resolution (WMD: − 9.9 days, 95% CI: − 19.1 to − 0.71, *I*^*2*^ = 96.7%, Fig. 5).

For diagnostic resolution, a sample size > 500 and less than 6-month follow-up length explained 94.5% of the heterogeneity. Excluding any single trial did not change our effect estimate. There was no evidence of publication bias (Egger’s test, *p* = 0.08). For time until diagnostic resolution, sample size and length of follow-up explained 86.1% and 91.5% of the heterogeneity. We found no evidence of publication bias (Egger’s test, *p* = 0.87).

## DISCUSSION

We found that patient navigators doubled the likelihood of screening for breast cancer. The impact of PN on diagnostic resolution was less clear; there was no improvement in resolution, but a reduction in the average time until resolution. This likely reflects the small number of trials; while the odds of diagnostic resolution did not quite meet statistical significance, it was nearly double in the PN group, reflecting inadequate power. The average number of days until resolution improved with PN, by nearly 10 days.

Recent studies have shown that PN improved mammography screening rates in medically underserved settings, and this effect may be especially pronounced for women who have not been previously screened.^3, 29^ We found that PN is effective in increasing mammography utilization among minority or underserved communities similar to other recent meta-analyses.^8, 9^ However, there was significant qualitative and quantitative heterogeneity with a great deal of variability in the design and implementation of the programs assessed. Additional studies would help clarify the significance and identify patients most likely to benefit from PN. To facilitate the aforementioned task, exploring the sources of heterogeneity and its significance is important.

Our findings suggest that follow-up time is one of the important sources of heterogeneity, which could be explained by the results of several previously published studies, demonstrating that longer trials demonstrated greater benefits, and shorter trials may underestimate the effectiveness of PN.^30, 31^ Trialists and policy makers should anticipate this lag when studying and implementing PN, as PN evidently takes time to achieve maximum benefit.^6, 30^ Other sources of heterogeneity, such as education and race, suggest that PN may be useful in helping certain patients navigate the health care system.

Reducing mortality rate through breast cancer screening will be incompletely realized if timely diagnostic follow-up for abnormal screening does not occur. We found a trend toward improved diagnostic resolution with a reduction in the number of days between the abnormal mammogram and final disposition. The paucity of trials limited our ability to provide definitive recommendations.

Our study has a number of limitations: first, most of the studies were from the USA; second, we identified evidence of publication bias as it is likely that small studies with negative results could remain unpublished; third, we found that PN doubled diagnostic resolution, an important clinical difference. The lack of statistical significance could be explained by the few included studies; our analysis lacked power. That we reduced the time but not the odds may also reflect differences in parametric and nonparametric tests in demonstrating statistical significance; finally, some studies combined data on the impact of PN on breast, cervical, and rectal screening, making it impossible to extract the data of the breast screening population.

Women needing breast cancer screening are a heterogeneous group, varying by menopausal status, age, education, and race. PN may be particularly useful in assuring screening and follow-up for vulnerable women. PN improves screening rates. Additional studies are required to assess the impact on diagnostic resolution, to determine which subgroups may benefit most and what types of PN interventions are most effective.

## Supplementary Information

ESM 1 (DOCX 13 kb)

## Data Availability

Data sharing is not applicable to this article as no datasets were generated or analyzed during the current study.
