# Supplementary material for: Impact of Patient Navigation on Population-Based Breast Screening: a Systematic Review and Meta-analysis of Randomized Clinical Trials
Source: J Gen Intern Med. 2022 Jun 1;37(11):2811–20. doi: 10.1007/s11606-022-07641-y (PMC9411406; doi:10.1007/s11606-022-07641-y)
Supplement: Supplementary file 1 — (DOCX 13 kb) [file 11606_2022_7641_MOESM1_ESM.docx]

**Supplementary Materials**

Search strategy for randomized controlled trials

Pubmed

#1：breast[all fields] or mammary [all fields]

#2: neoplasms [Mesh]

#3: neoplasm[all fields] or cancer[all fields] or tumor[all fields] or carcinoma[all fields] or malignancy[all fields]

#4: #2 OR #3

#5: #1 AND #4

#6：Patient Navigation [Mesh]

#7: nurse navigator[all fields] OR patient navigations[all fields] OR patient navigators[all fields] OR patient navigator[all fields]

#8:#6 OR #7

#9:"Randomized Controlled Trial" [Publication Type]

#10: #5 AND #8 AND #9

Embase

#1：breast OR mammary

#2: 'neoplasm'/exp OR neoplasms OR malignancy OR cancer OR tumor OR carcinoma

#3: #1 and #2

#4: nurse AND navigator OR (patient AND navigations) OR (patient AND navigators) OR (patient AND navigator) OR(patient AND navigation)

#5: 'randomized controlled trial'/exp

#6: #3 AND #4 AND #5

Web of Science

#1：theme: (breast) OR theme: (mammary)

Databases= WOS, BIOSIS, CSCD, DIIDW, INSPEC, KJD, MEDLINE, RSCI, SCIELO time span=all years

#2:theme: (neoplasm) OR theme: (cancer)  OR theme: (tumor) OR theme: (carcinoma) OR theme: (malignancy)  OR theme: (neoplasms)

Databases= WOS, BIOSIS, CSCD, DIIDW, INSPEC, KJD, MEDLINE, RSCI, SCIELO time span=all years

#3：#2 AND #1

#4：theme: (nurse navigator) OR theme: (patient navigations) OR theme: (patient navigators) OR theme: (patient navigator) OR theme: (patient AND navigation)

Databases= WOS, BIOSIS, CSCD, DIIDW, INSPEC, KJD, MEDLINE, RSCI, SCIELO time span=all years

#5：theme: (randomized controlled trial)

#6: #3 AND #4 AND #5

Cochrane Library

#1：(breast)[all text] or (mammary)[all text]

#2: neoplasms [Mesh]

#3: neoplasm[all text] OR cancer[all text] OR tumor[all text] OR carcinoma[all text] OR malignancy[all text]

#4: #2 OR #3

#5: #1 AND #4

#6：patient navigation [Mesh]

#7: nurse navigator[all text] OR patient navigations[all text] OR patient navigators[all text] OR patient navigator[all text]

#8:#6 OR #7

#9: randomized controlled trial[Mesh]

#10: randomized controlled trial[all text]

#11: #9 OR #10

#12: #5 AND #8 AND #11
